# Gastrointestinal Bleeding After Percutaneous Coronary Intervention: Incidence, Risk Factors, and Outcomes

**DOI:** 10.7759/cureus.70248

**Published:** 2024-09-26

**Authors:** Ahmed Al-Hatmi, Marwa Al-Habsi, Malik Al-Ghafri, Raiyan Al-Siyabi, Rinad Al-Ruheili, Sunil K Nadar, Said A Al-Busafi

**Affiliations:** 1 Internal Medicine Training Program, Oman Medical Specialty Board, Muscat, OMN; 2 Clinical Physiology, Sultan Qaboos University Hospital, Muscat, OMN; 3 College of Medicine and Health Sciences, Sultan Qaboos University, Muscat, OMN; 4 Cardiology, Sultan Qaboos University Hospital, Muscat, OMN; 5 Gastroenterology Unit, Department of Medicine, College of Medicine and Health Sciences, Sultan Qaboos University, Muscat, OMN

**Keywords:** acute gastrointestinal bleed, antiplatelet agents, peptic ulcer diseases, primary percutaneous coronary intervention (pci), risk factors

## Abstract

Background: Percutaneous coronary intervention (PCI) is a well-established method for treating coronary artery disease. However, stent implantation necessitates the use of antiplatelet agents, which increases the risk of gastrointestinal bleeding (GIB). This study aims to assess the incidence, etiology, and outcomes of GIB within one year post-PCI at Sultan Qaboos University Hospital (SQUH), Muscat, Oman.

Methods: This retrospective analysis included 435 patients who underwent PCI at SQUH between January and December 2017, excluding those with multiple PCI procedures and incomplete data. GIB was defined using the thrombolysis in myocardial infarction (timi) bleeding score. Statistical analysis was performed using IBM SPSS Statistics for Windows, version 23 (IBM Corp., Armonk, NY) with binary logistic regression employed to identify significant risk factors for GIB. A p-value < 0.05 was considered statistically significant.

Results: GIB was identified in 15 patients (age 67.8 ± 8.8 years; 60% male), resulting in an incidence within one year post-PCI of 3.4%. Melena (53.3%) and hematemesis (20.0%) were the most frequent clinical presentations, with peptic ulcer disease being the predominant underlying etiology (40%). Significant risk factors included advanced age, chronic kidney disease, pre-existing peptic ulcer disease, and the use of non-steroidal anti-inflammatory drugs.

Conclusions: The incidence of GIB observed in this study aligns with previously reported rates. These findings highlight the importance of pre-PCI risk stratification for bleeding, especially in high-risk patients. Prophylactic measures, such as the use of gastric protective agents, should be considered to mitigate the risk of GIB in patients undergoing PCI.

## Introduction

Percutaneous coronary intervention (PCI) is a widely adopted and effective therapeutic approach for managing ischemic heart disease, particularly in patients with acute coronary syndromes and significant coronary artery stenosis [1]. The procedure, which involves the insertion of stents to maintain coronary artery patency, has significantly reduced the morbidity and mortality associated with coronary artery disease (CAD) by improving myocardial perfusion and alleviating symptoms like angina [2]. However, PCI is not without its risks, with gastrointestinal bleeding (GIB) being one of the most concerning complications. This complication is primarily linked to the mandatory use of dual antiplatelet therapy (DAPT) [3]. DAPT, typically consisting of aspirin and a P2Y12 inhibitor, is essential to prevent stent thrombosis. Patients who undergo PCI with stent placement require long-term antiplatelet therapy, typically with dual antiplatelet therapy for one year, followed by lifelong single-agent therapy [1]. Despite its benefits in lowering ischemic risk, DAPT increases the likelihood of both major and minor bleeding events, including GIB, which is associated with heightened morbidity and mortality [4,5].

The incidence of post-PCI bleeding reported in the literature ranges from 0.8% to 11.5%, with GIB accounting for a significant portion of these cases [6]. The variation in GIB incidence, reported between 0.6% and 4%, depends on factors such as the population studied, bleeding definitions, and follow-up duration [7]. The occurrence of GIB after PCI not only increases morbidity and hospital stay duration but also elevates in-hospital mortality rates [8]. Most of these bleeding complications originate from the upper gastrointestinal tract and may necessitate discontinuation of DAPT, thereby increasing the risk of stent thrombosis and its associated complications.

The etiology of GIB in PCI patients often involves a multifactorial interplay of pre-existing gastrointestinal conditions, such as peptic ulcer disease, concomitant use of medications like non-steroidal anti-inflammatory drugs (NSAIDs), and patient-specific factors, including advanced age and chronic kidney disease [9]. Given the severity of GIB and its impact on patient outcomes, it is crucial to identify high-risk patients and implement preventive strategies, such as prescribing gastric protective agents and carefully selecting antiplatelet regimens. Despite the known risks, there remains a lack of consensus on the optimal approach to managing GIB risk in PCI patients, particularly in balancing effective antithrombotic therapy with bleeding risks.

This study aims to fill this gap by assessing the incidence, risk factors, and outcomes of GIB within one year post-PCI at Sultan Qaboos University Hospital (SQUH), Muscat, Oman. By analyzing data from a real-world clinical setting, we hope to contribute to the body of evidence guiding clinical decision-making in this challenging aspect of post-PCI care. Although GIB is recognized as a significant complication following PCI, its nature and etiology are often not fully elucidated in existing trials. Furthermore, there is a paucity of studies investigating post-PCI GIB in the Middle East. Understanding these bleeding events is crucial for their prevention, making this study essential for assessing the incidence, etiology, and outcomes of GIB within one year after PCI at our institution.

## Materials and methods

This retrospective cohort study was conducted following the Strengthening the Reporting of Observational Studies in Epidemiology (STROBE) guidelines [10]. The study involved adult patients (aged 18 years and older) who underwent percutaneous coronary intervention (PCI) at Sultan Qaboos University Hospital (SQUH) between January and December 2017. Ethical approval for this study was obtained from the Medical Research Ethics Committee at the College of Medicine and Health Sciences, SQUH (Approval No. 1089, dated May 4, 2015).

The institutional database identified 547 patients who underwent PCI during the study period. Inclusion criteria comprised all adult patients (18 years and older) with complete clinical data. Exclusion criteria included patients with incomplete data, those who could not be followed up within one year post-PCI, patients undergoing coronary artery bypass grafting (CABG), or those receiving conservative treatment without PCI. After applying these criteria, 435 patients were included in the final analysis. For patients with multiple PCI procedures during the study period, only the first intervention was considered the index admission to avoid overrepresentation of individuals.

Baseline laboratory tests were conducted for all patients immediately following PCI, including renal function tests such as glomerular filtration rate (GFR), creatinine, and liver function tests (LFTs). For patients who experienced GIB, the last available set of laboratory results before the bleeding episode was used to evaluate whether abnormal values, particularly renal and liver dysfunction, could be considered risk factors for GIB.

GIB was defined based on clinical events documented by a physician, such as coffee-ground emesis, hematemesis, melena, or hematochezia, or based on endoscopic evidence of active bleeding within the gastrointestinal tract (upper or lower). GIB events were classified using the thrombolysis in myocardial infarction (TIMI) bleeding criteria. Non-CABG-related TIMI major bleeding was defined as any intracranial bleeding, a hemoglobin (Hb) drop greater than 5 g/dL, or any bleeding that resulted in death within seven days of the event. TIMI minor bleeding was classified as overt bleeding with a 3-5 g/dL drop in Hb, while TIMI minimal bleeding events did not meet the criteria for major or minor bleeding.

Data were analyzed using IBM SPSS Statistics for Windows, version 23 (IBM Corp., Armonk, NY). Continuous variables were expressed as mean with standard deviation (SD) or median with interquartile range (IQR), depending on the normality of the distribution. Categorical variables were presented as numbers with percentages. The differences between groups with and without GIB were evaluated using the Student’s t-test for continuous variables and the chi-square test for categorical variables. Binary logistic regression analysis was performed to identify predictors of GIB, with the presence of bleeding as the dependent variable. A p-value < 0.05 was deemed statistically significant in all analyses. Sensitivity analyses were conducted to assess the impact of key variables on the outcomes and to ensure the robustness of the findings.

## Results

A total of 435 patients (mean age 60.6 ± 12.3 years; 74.7% male) were included in the final analysis. GIB occurred in 15 patients (mean age 67.8 ± 8.8 years; 60% male) within one year of the PCI, yielding an incidence rate of 3.4%. The median time to bleeding was 17 days (range: 2-205 days), with most cases (53.3%) occurring within the first month. Blood transfusions were required for seven patients (46.7%), with five classified as major and two as minor bleeds. The remaining eight patients experienced minimal bleeding, as determined by the TIMI bleeding scale.

Table 1 summarizes the demographic features of patients with and without GIB. Patients with GIB were older (mean age 67.8 ± 8.8 years) than those without GIB (mean age 60.3 ± 12.3 years; p = 0.02). No significant difference in gender distribution was observed between the groups. Both groups had a similar proportion of patients on dual antiplatelet therapy. However, nearly all patients in the GIB group were on proton pump inhibitors (PPI) (93.3% vs. 52.3%; p = 0.007) and non-steroidal anti-inflammatory drugs (100% vs. 46.9%; p = 0.001) prior to the GIB incidence. Traditional cardiovascular risk factors did not differ significantly between the groups. However, the GIB group exhibited a higher prevalence of chronic kidney disease (33.3% vs. 8.5%; p = 0.05), a history of prior PCI (40% vs. 12.8%; p = 0.01), lower glomerular filtration rate (median 67 ml/min vs. 80 ml/min; p = 0.01), and higher creatinine levels (median 102 µmol/l vs. 80 µmol/l; p = 0.05). Additionally, the GIB group had a higher proportion of patients with abnormal liver function tests (60% vs. 20.9%; p = 0.002) and pre-existing peptic ulcer disease (26% vs. 0.7%; p < 0.001).

**Table 1:** Demographic and clinical characteristics of patients with and without gastrointestinal bleeding post-percutaneous coronary intervention PPI, proton pump inhibitor; H2RB, histamine2 receptor blocker; PCI, percutaneous coronary intervention; CABG, coronary artery bypass grafting; CKD, chronic kidney disease; GFR, glomerular filtration rate Figures are number (%), mean (standard deviation), or median (interquartile range). Analysis by chi-square test, except *Student's t-test and **Mann-Whitney U test

	Without bleeding (n=420)	With bleeding (n=15)	p-value
Age (years)	60.3 + 12.3	67.8 + 8	0.02*
Sex			
Male	316 (75.2%)	9 (60%)	0.18
Female	104 (24.8%)	6 (40%)	
Concomitant medications			
Aspirin	418 (99.5%)	15 (100%)	0.9
Clopidogrel	413 (98.3%)	15 (100%)	0.91
Dual antiplatelet	413 (98.3%)	15 (100%)	0.91
PPI	220 (52.3%)	14 (93.3%)	0.007
H_2_RB	54 (12.8%)	4 (26.6%)	0.006
NSAIDs	197 (46.9%)	15 (100%)	0.001
Warfarin	25 (5.9%)	1 (6.6%)	0.91
Steroids	9 (2.1%)	1 (6.6%)	0.31
Diabetes	224 (53.3%)	10 (66.6%)	0.9
Hypertension	262 (62.3%)	13 (86.6%)	0.28
Smoking status			
Current	86 (20.4%)	2 (13.3%)	0.6
Ex-smoker	23 (5.4%)	0	
Hyperlipidemia	171 (40.7%)	7 (46.6%)	0.8
Previous PCI	54 (12.8%)	6 (40%)	0.01
Previous CABG	18 (4.2%)	2 (13.3%)	0.2
CKD	36 (8.5%)	5 (33.3%)	0.05
History of Peptic ulcer	3 (0.7%)	4 (26.6%)	<0.001
Abnormal liver functions	88 (20.9%)	9 (60%)	0.002
GFR (ml/min)	80 (63-90)	67 (20-83)	0.01**
Creatinine (µmol/l)	80 (60-99)	102 (61-214)	0.05**
Mortality at one year	10 (2.3%)	4 (26.6%)	<0.001

The clinical profiles of patients who experienced GIB are presented in Table 2. Melena was the most common presentation (53.3%), followed by hematemesis (33.3%) and hematochezia (13.3%). Endoscopic examinations were performed on 10 patients with GIB. Two patients were too ill for invasive procedures and died before intervention, while another three patients refused the procedure. Endoscopic findings revealed that four patients (40%) had peptic ulcers, three (30%) had hemorrhagic gastritis, two had submucosal petechiae, and one had a gastric polyp. Therapeutic injections were used to control bleeding in all patients with peptic ulcers. Two patients with erosive gastritis experienced re-bleeding during hospitalization. Among the GIB group, four patients (26.6%) died within the first year post-PCI, compared to 10 deaths in the non-GIB group during the same period.

**Table 2:** Clinical presentations and endoscopic findings in patients with gastrointestinal bleeding post-percutaneous coronary intervention

	Number (%)
Presentation	
Hematochezia	2 (13.3%)
Malena	8 (53.3%)
Hematemesis	5 (33.3%)
Gastroscopic findings (n=10)	
Peptic ulcer	4 (40%)
Hemorrhagic gastritis	3 (30%)
Polyp	1 (10%)
Submucosal petechiae	2 (20%)

Binary logistic regression identified pre-existing peptic ulcer disease as the strongest predictor of GIB (odds ratio {OR} 12.5, 95% CI 2.1-74.8; p < 0.001), followed by chronic kidney disease (OR 4.8, 95% CI 1.72-13.3; p = 0.001) and previous PCI (OR 3.12, 95% CI 1.6-6.06; p = 0.005). Additional significant factors included creatinine levels greater than 120 µmol/l, glomerular filtration rate (GFR) less than 60 ml/min, and age greater than 60 years (Table 3).

**Table 3:** Predictors and results of binary logistic regression analysis for gastrointestinal bleeding post-percutaneous coronary intervention PUD, peptic ulcer disease; GFR, glomerular filtration rate; CKD, chronic kidney disease; PCI, percutaneous coronary intervention; LFT, liver function test

	Odds ratio	95% confidence levels	p-value
Pre-existing PUD	12.5	2.1-74.8	<0.001
GFR <60 ml/min	1.96	1.1-3.4	0.03
Pre-existing CKD	4.8	1.72-13.3	0.001
Previous PCI	3.12	1.6-6.06	0.005
Creatinine >120 µmol/l	3.78	1.2-10.8	0.01
Abnormal LFT	2.4	1.2-2.08	0.02
Age >60 years	1.58	1.2-2.08	0.02

## Discussion

Bleeding remains a significant concern after coronary intervention, ranging from minor access site hematomas to severe and potentially fatal intracranial or gastrointestinal bleeds. In early coronary intervention trials, bleeding was often viewed as acceptable collateral damage due to the substantial reduction in ischemic events with potent antiplatelet agents. It was not included in primary outcome data [11]. However, larger trials have since demonstrated that bleeding associated with PCI is linked to higher morbidity and mortality, underscoring the need to address it [12,13]. The National Cardiovascular Data Registry in the US reports that major bleeding complications account for 12.1% of all in-hospital mortality after PCI [14]. Consequently, later trials have appropriately included bleeding complications as part of composite primary endpoints [15,16]. Our study supports this, showing a higher mortality rate among patients with GIB compared to those without.

Access site-related bleeding complications are the most common after PCI. However, these complications have significantly decreased with the shift from a femoral to a radial artery approach in most centers [17]. Among non-access site bleeding complications, GIB is the most prevalent form of major bleeding [5,12,18].

Identifying at-risk patients and initiating preventive measures for GIB post-PCI is crucial. A recent large study from the British Cardiovascular Interventional Database demonstrated that GIB impacts mortality up to a year after PCI [19]. Several mechanisms explain how GIB can increase mortality post-PCI. Significant blood loss may cause hemodynamic compromise, worsening myocardial ischemia, or causing renal injury due to hypoperfusion. Additionally, temporary interruption of antiplatelet therapy due to bleeding increases the risk of stent thrombosis and subsequent myocardial infarction.

This study is the first retrospective analysis of GIB post-PCI in Oman. The incidence of GIB in our study (3.4%) aligns with rates reported in the literature, which range from 0.4% to 3.4% [8,20,21]. Our findings are also consistent with existing studies, which indicate that the majority of bleeds occur within the first-month post-PCI [8,20,21]. While most studies identify hematemesis as the most common presentation of GIB [8,18,19,21], our study found that melena was the most frequent. Although hematemesis was also common in our study, we do not have a clear explanation for the higher incidence of melena.

In our study, the risk factors for bleeding included age, concomitant NSAID use, pre-existing peptic ulcer disease, renal disease, abnormal liver function tests, and elevated creatinine levels. These findings are consistent with previous studies that have identified similar risk factors, including advanced age, acute presentation, renal failure, heart failure, and hemodynamic compromise [4,13]. Pre-existing peptic ulcer disease, concomitant NSAID use, or the use of other anticoagulants are also well-known risk factors for GIB, which our study confirms.

Regarding etiology, a study focusing on upper GIB post-PCI identified gastric erosions (20.2%), duodenal ulcers (14.3%), esophagitis (13.1%), Mallory-Weiss tears (13.1%), duodenitis (7.1%), and esophageal erosions (7.1%) as the most common causes of bleeding [18]. In our study, bleeding ulcers were the most frequent cause of GIB, and all affected patients received therapeutic injections for their ulcers. We strongly recommend that patients with GIB undergo upper GI endoscopy as part of their management.

Almost all patients in our study who developed GIB had already been started on PPI after the procedure. Many had a history of peptic ulcer disease and may have been on long-term PPI therapy. For others without a history of peptic ulcer disease, the initiation of PPI may reflect their identification as high-risk for bleeding. The use of PPI with clopidogrel is a controversial issue sparked by retrospective studies suggesting that PPIs may reduce clopidogrel's efficacy [22]. It was hypothesized that PPIs inhibit the CYP450 enzyme responsible for generating clopidogrel’s active metabolite, thus reducing its effectiveness and increasing the risk of major cardiac events. However, a prospective trial confirmed that PPIs reduce GI bleeding rates in patients on dual antiplatelet therapy (1.1% vs. 2.9%; hazard ratio {HR}: 0.34; p < 0.001) without increasing major cardiac events [23]. Current guidelines recommend using a PPI with dual antiplatelet therapy in patients at high risk for GIB [1].

Recent trials have focused on the choice and duration of antiplatelet agents to minimize the risk of GIB. Studies involving newer-generation drug-eluting stents have demonstrated the safety of shorter durations of dual antiplatelet therapy in high bleeding-risk patients without an increase in ischemic events [24,25]. Several studies comparing the gastrointestinal effects of aspirin and clopidogrel have yielded conflicting results [3]. Earlier trials suggested that aspirin was associated with higher GIB rates, but this is not entirely clear. In the CAPRIE (Clopidogrel Versus Aspirin in Patients at Risk of Ischemic Events) trial, involving 19,185 patients with vascular disease, those randomized to aspirin had a statistically higher risk of bleeding (0.72% vs. 0.52%; p < 0.05) [26]. However, the aspirin dose in this trial was 325 mg, not the currently recommended 75 mg, and the number needed to harm (NNH) was 500, suggesting the bleeding differences may not be as significant as once thought. A recent meta-analysis comparing aspirin with a P2Y12 inhibitor also found higher GIB rates with aspirin (1.56% vs. 1.22%; OR: 1.69; 95% CI: 1.12-2.56) [27]. However, this corresponds to an NNH of 152, suggesting that, in practice, these differences between aspirin and P2Y12 inhibitors are minor.

A recent study by Han et al., using magnetically controlled capsule endoscopy, revealed that nearly all patients on antiplatelet therapy developed some form of gastrointestinal mucosal injury, although overt bleeding was rare [28]. They also found that single antiplatelet therapy caused less injury than dual antiplatelet therapy and observed no difference in injury caused by aspirin versus clopidogrel.

Newer antiplatelet agents, such as ticagrelor and prasugrel, are associated with lower ischemic events than clopidogrel but carry a higher bleeding risk [29]. The concomitant use of anticoagulants in patients with atrial fibrillation or venous thromboembolism is another strong risk factor for bleeding. Our study did not include patients on ticagrelor or prasugrel; the number of patients on warfarin was too low to assess its effect. When assessing the balance between risk and benefit, these factors must be carefully considered before proceeding with coronary intervention in patients with a high bleeding risk [30].

This study has several limitations. As a retrospective study, it relies on clinical records, making it dependent on the quality of documentation. Some patients may have been treated for GIB at other hospitals without reporting it upon return to our facility. Additionally, this study was conducted at a single center with a small sample size. Despite these limitations, this is the first study of GIB in Oman post-PCI. Further prospective studies involving other interventional centers in Oman are needed to understand the true incidence and nature of GIB after coronary intervention in the country.

## Conclusions

This study highlights the significant yet often overlooked complication of GIB following PCI. With an incidence rate of 3.4%, GIB represents a substantial clinical concern, particularly given its association with increased mortality. The identification of risk factors such as advanced age, chronic kidney disease, pre-existing peptic ulcer disease, and NSAID use underscores the need for careful patient assessment and risk stratification before PCI. The findings emphasize the importance of proactive management strategies, including using gastric protective agents like PPIs and the judicious selection of antiplatelet therapy, to mitigate the risk of GIB. While our study provides valuable insights into the incidence and risk factors of GIB post-PCI, it also acknowledges the limitations inherent in its retrospective design and single-center scope. Future prospective studies across multiple centers in Oman are essential to confirm these findings and to refine further strategies for preventing and managing GIB in this high-risk population.
